# Partial substitution of red meat or processed meat with plant-based foods and the risk of colorectal cancer

**DOI:** 10.1007/s10654-024-01096-7

**Published:** 2024-01-23

**Authors:** Rilla Tammi, Niina E Kaartinen, Kennet Harald, Mirkka Maukonen, Heli Tapanainen, Stephanie A Smith-Warner, Demetrius Albanes, Johan G Eriksson, Pekka Jousilahti, Seppo Koskinen, Maarit A Laaksonen, Sanna Heikkinen, Janne Pitkäniemi, Anne-Maria Pajari, Satu Männistö

**Affiliations:** 1 Department of Public Health and Welfare, Finnish Institute for Health and Welfare (THL), P.O. Box 30, Helsinki, 00271 Finland; 2 Departments of Nutrition and Epidemiology, Harvard T.H. Chan School of Public Health, Boston, MA USA; 3 Division of Cancer Epidemiology and Genetics, National Cancer Institute, National Institutes of Health (NIH), Bethesda, MD USA; 4 Department of General Practice and Primary Health Care, University of Helsinki and Helsinki University Hospital, Helsinki, Finland; 5 Folkhälsan Research Center, University of Helsinki, Helsinki, Finland; 6 Department of Obstetrics and Gynaecology and Human Potential Translational Research programme, Yong Loo Lin School of Medicine, National University of Singapore, Singapore, Singapore; 7 Agency for Science, Technology and Research, Singapore Institute for Clinical Sciences (SICS), A*STAR, Singapore, Singapore; 8 School of Public Health, Faculty of Medicine, University of Sydney, Sydney, NSW Australia; 9 Finnish Cancer Registry, Institute for Statistical and Epidemiological Cancer Research, Helsinki, Finland; 10 Health Sciences Unit, Faculty of Social Sciences, Tampere University, Tampere, Finland; 11 Department of Public Health, University of Helsinki, Helsinki, Finland; 12 Department of Food and Nutrition, University of Helsinki, Helsinki, Finland

**Keywords:** Epidemiology, Fruit, Nutrition, Sustainability, Vegetable, Whole grain

## Abstract

**Objectives:**

Shifting from animal-based to plant-based diets could reduce colorectal cancer (CRC) incidence. Currently, the impacts of these dietary shifts on CRC risk are ill-defined. Therefore, we examined partial substitutions of red or processed meat with whole grains, vegetables, fruits or a combination of these in relation to CRC risk in Finnish adults.

**Methods:**

We pooled five Finnish cohorts, resulting in 43 788 participants aged ≥ 25 years (79% men). Diet was assessed by validated food frequency questionnaires at study enrolment. We modelled partial substitutions of red (100 g/week) or processed meat (50 g/week) with corresponding amounts of plant-based foods. Cohort-specific hazard ratios (HR) for CRC were calculated using Cox proportional hazards models and pooled together using random-effects models. Adjustments included age, sex, energy intake and other relevant confounders.

**Results:**

During the median follow-up of 28.8 years, 1124 CRCs were diagnosed. We observed small risk reductions when red meat was substituted with vegetables (HR 0.97, 95% CI 0.95 − 0.99), fruits (0.97, 0.94 − 0.99), or whole grains, vegetables and fruits combined (0.97, 0.95 − 0.99). For processed meat, these substitutions yielded 1% risk reductions. Substituting red or processed meat with whole grains was associated with a decreased CRC risk only in participants with < median whole grain intake (0.92, 0.86 − 0.98; 0.96, 0.93 − 0.99, respectively; *p*_interaction_=0.001).

**Conclusions:**

Even small, easily implemented substitutions of red or processed meat with whole grains, vegetables or fruits could lower CRC risk in a population with high meat consumption. These findings broaden our insight into dietary modifications that could foster CRC primary prevention.

**Supplementary Information:**

The online version contains supplementary material available at 10.1007/s10654-024-01096-7.

## Introduction

Promoting healthier and more sustainable food consumption requires a global shift towards more plant-based diets [1]. Current Western diets, which are high in energy and animal-based foods, contribute heavily to the growing global burden of chronic diseases and environmental degradation. The detrimental effects on health and the environment have been attributed particularly to high red and processed meat consumption [2–6]. Hence, reducing their consumption has been highlighted in different international strategies [1, 7] and dietary recommendations [8–12]. In contrast, increasing the consumption of plant-based foods, such as whole grains, vegetables, fruits and legumes, has been recommended because of their associations with beneficial health effects [3, 4, 13–15] and low greenhouse gas emissions [2]. As in other Western countries [16], in Finland, red and processed meat consumption exceeds recommendations, especially in men, while the consumption of plant-based foods remains insufficient [17].

Strong evidence indicates that consumption of red meat and processed meat are associated with an increased risk of colorectal cancer (CRC) [6, 18]. In 2020, CRC was the third most common cancer in the world with 1.9 million new cases diagnosed [19]. The International Agency for Research on Cancer (IARC) has classified red meat as probably carcinogenic and processed meat as carcinogenic, mainly based on evidence of increased CRC risk [6]. Similarly, the World Cancer Research Fund (WCRF) recommends limiting red and processed meat consumption as red meat is considered a probable and processed meat a convincing cause of CRC [18]. In the meta-analyses conducted by the WCRF and the American Institute for Cancer Research (AICR), a 100 g/d increase in red meat consumption was associated with a 12% higher CRC risk and a 50 g/d increase in processed meat consumption with an 18% higher risk [13]. In contrast, the WCRF/AICR reported a 17% decrease in CRC risk for each 90 g/d increase in whole grain intake. A small 2% risk reduction also occurred with a 100 g/d increase in vegetable consumption. The WCRF/AICR meta-analyses included 6 − 11 cohort studies with 6000 − 14 000 CRC cases [13].

While the need for reducing red and processed meat consumption and eating more plant-based diets is evident, the implications of substituting red and processed meat with plant-based foods on CRC risk are ill-defined. Based on the displayed associations between single food groups and CRC risk, substituting red and processed meats with plant-based foods would presumably decrease CRC risk [13]. The total impact of a dietary shift on disease risk may not, however, directly reflect the summed impacts of single food groups. Moreover, the associations may vary depending on which plant-based foods are substituted for animal-based foods.

To date, few studies have assessed CRC risk in relation to dietary shifts towards more plant-based diets, and the focus has been on shifting sources of protein intake. A large cohort study including 490 000 US Americans and 9000 incident CRC cases modelled substitutions of total protein intake from red meat with plant-based protein and reported 11% lower CRC risk in the highest substitution quintile compared with the lowest quintile [20]. In an Italian cohort study (*n* = 45 000, 538 incident CRC cases), substituting 3% of energy intake from red and processed meat-derived protein with plant-based protein was associated with a decreased rectal cancer risk [21]. The substitution was also associated with an increased colon cancer risk, but this association was driven by plant-based proteins from foods with high glycaemic index.

The previous studies have not examined substitutions of red meat and processed meat separately in relation to CRC, even though processed meat seems to be a stronger risk factor for CRC than red meat is [6]. Similarly, no study has explored substitutions of red meat or processed meat with whole grains, vegetables, fruits or legumes in relation to CRC risk. Consequently, we aimed to examine partial substitutions of red meat (100 g/week) or processed meat (50 g/week) with corresponding amounts of whole grains, vegetables, fruits, legumes or a combination of these in relation to CRC risk in Finnish adults. We modelled moderate substitutions of 100 g or 50 g per week to explore dietary changes that would be easy to implement and maintain in real-life settings.

## Methods

### Participants

Our data comprised participants from five cohort studies of Finnish adults conducted at the Finnish Institute for Health and Welfare: the Alpha-Tocopherol Beta-Carotene Cancer Prevention Study (ATBC) [22], the Health 2000 Health Examination Survey (Health 2000) [23], the Helsinki Birth Cohort Study (HBCS) [24], the DIetary, Lifestyle and Genetic Determinants of Obesity and Metabolic Syndrome 2007 Study (DILGOM 2007) [25], and the National FINRISK 2012 Study (FINRISK 2012) [26] (Table 1). ATBC was initially a trial studying alpha-tocopherol and beta-carotene supplementation and cancer risk in middle-aged male smokers. HBCS aimed to assess the impacts of early growth on health later in life in middle-aged individuals. Health 2000, DILGOM 2007 (conducted within the National FINRISK 2007 Study) and FINRISK 2012 were national health monitoring surveys. All cohorts included a health examination and self-administered questionnaires, such as the food frequency questionnaire (FFQ). Each cohort was followed using national health registers.

**Table 1:** Cohorts included in the pooled analyses of partial substitutions of red meat or processed meat with plant-based foods and the risk of colorectal cancer (CRC)

Cohort	Baseline years	Baseline age, years	Final number of participants^a^	Number of men (%)	Median follow-up time, years	Number of incident CRC cases
ATBC (22)	1984–1988	50–70	26 944	26 944 (100%)	29.9	949
Health 2000 (23)	2000–2001	30–99	5736	2575 (45%)	15.2	70
HBCS (24)	2001–2004	56–69	1862	875 (47%)	12.1	36
DILGOM 2007 (25)	2007	25–74	4685	2175 (46%)	12.6	46
FINRISK 2012 (26)	2012	25–74	4561	2081 (46%)	7.8	23
Total			43 788	34 650 (79%)	28.8	1124

ATBC, the Alpha-Tocopherol, Beta-Carotene Cancer Prevention Study; Health 2000, the Health 2000 Health Examination Survey; HBCS, the Helsinki Birth Cohort Study; DILGOM 2007, the DIetary, Lifestyle and Genetic Determinants of Obesity and Metabolic Syndrome 2007 Study; FINRISK 2012, the National FINRISK 2012 Study

^a^Exclusion criteria: missing or inadequately filled FFQ (incomplete questionnaire with several empty rows, exclusions made case by case), implausible energy intake (ATBC: energy intake < 1000 or > 5000 kcal/d; Health 2000: energy intake < 600 or > 7000 kcal/d; HBCS, DILGOM 2007 and FINRISK 2012: 0.5% sex-specific extremes in energy intake distribution) and history of cancer other than non-melanoma skin cancer at baseline

In each cohort, participants were excluded based on implausible energy intake (ATBC: energy intake < 1000 or > 5000 kcal/d; Health 2000: energy intake < 600 or > 7000 kcal/d; HBCS, DILGOM 2007 and FINRISK 2012: 0.5% sex-specific extremes in energy intake distribution), missing or inadequately filled FFQ (incomplete questionnaire with several empty food item rows, exclusion made case by case) or a history of cancer other than non-melanoma skin cancer at baseline (Online Resource 1). After the exclusions, our final study sample comprised 43 788 participants (ATBC *n* = 26 944, Health 2000 *n* = 5736, HBCS *n* = 1862, DILGOM 2007 *n* = 4685, FINRISK 2012 *n* = 4561).

This study was conducted according to the guidelines laid down in the Declaration of Helsinki. Each cohort followed the ethical standards in effect at the time of the study. For instance, in the later cohorts, the Ethics Committee of the Hospital District of Helsinki and Uusimaa approved all procedures involving human subjects. All participants provided written informed consent.

### Dietary assessment

Information on dietary intake was gathered by validated, semi-quantitative FFQs covering habitual food consumption over the past year [27–30]. The inquired foods included the most used foods in Finland based on the National FinDiet Surveys conducted since 1982 [17]. In ATBC, the FFQ included 276 food items and mixed dishes. The usual consumption frequency of each item and dish was recorded according to daily, weekly or monthly consumption, based on 3 − 5 portion sizes depicted in a portion size picture booklet. The participants completed the FFQ at home and checked it with a trained study nurse during the health examination. An FFQ including approximately 130 food items and mixed dishes was used in the remaining cohorts [23–27]; the item number varied slightly across studies due to regular updates on the FFQ based on the National FinDiet Surveys [17]. The consumption of each item was recorded by up to nine frequency categories (from ‘never or seldom’ to ‘at least six times a day’) and fixed portion sizes. The participants completed the FFQ either at the study site (HBCS, DILGOM 2007) or at home and mailed it to the Finnish Institute for Health and Welfare (Health 2000, FINRISK 2012).

Based on the FFQs, the average daily consumption of foods (g/d) and intake of energy (kJ/d) and nutrients (g/d) were calculated using the Finnish Food Composition Database Fineli® and an in-house software [31]. Within the calculation processes, all mixed dishes were decomposed into their ingredients (e.g., uncooked red meat; rye flour and rolled oats; processed meat, vegetables, fruits and legumes as such;) based on standard recipes in the database. The daily consumptions were multiplied by seven to calculate weekly consumptions.

We modelled changes in the consumption of red meat, processed meat, whole grains, vegetables, fruits and legumes. We studied red meat and processed meat separately because of stronger evidence of causality between processed meat and CRC risk than red meat and CRC risk [18]. In delineating the food groups, we used the food classifications of Fineli® [31]. Red meat included beef, pork and lamb (e.g., minced meat, beef steak) (Online Resource 2). Processed meat comprised sausages (e.g., fresh sausages, bratwurst) and cold cuts (e.g., smoked ham, meat sausages, meat cuts), including those made of beef, pork or lamb. Whole grain intake was assessed based on the combined consumption of rye, oat and barley as in our previous study, we demonstrated that this combination corresponds well (*r* = 0.99) to total whole grain intake among Finnish adults [32]. Vegetables included all vegetables (excluding legumes and potatoes) as well as nuts and seeds, of which consumption is low among Finnish adults [17]. We studied legumes as their own food group for their high content of high-quality plant protein and because they are often considered alternatives for animal-based protein sources. Legumes comprised all legumes commonly used in Finland, such as green peas, soya, and beans in their different forms. Fruits included all fruits and berries.

#### Colorectal cancer ascertainment

We obtained information on incident CRC cases (ICD-10 codes C18, C19.9 and C20.9 or ICD-9 codes 153, 154.0, 154.1) from the Finnish Cancer Registry, which upholds high-quality and comprehensive nationwide records on all cancer cases diagnosed in Finland since 1953. The coverage of colorectal and anal cancers was 97% [33]. The registry uses personal identity codes issued to all Finnish citizens and permanent residents in linking cancer diagnoses to individuals.

The follow-up started from the date of enrolment and continued until CRC diagnosis, death or the end of follow-up. The follow-up ended in ATBC on 31 December 2016; in Health 2000 on 31 December 2015; in HBCS on 31 December 2014; and in DILGOM 2007 and FINRISK 2012 on 31 December 2019.

#### Sociodemographic factors, lifestyle factors and anthropometric measures

Information on participants’ sex and age originated from the sampling frame (Finnish Population Information System). Sociodemographic and lifestyle factors were recorded by self-administered questionnaires inquiring into, for example, education, leisure time physical activity and smoking habits. The self-administered questionnaires also included questions for women on the use of hormone replacement therapy (HRT). These variables, along with dietary factors, were harmonized between the cohorts. Information on the family history of CRC (first-degree relatives) was only gathered in ATBC.

We categorised participants into those with low, middle or high educational attainment according to the thirds of self-reported years of education, taking into account sex and birth year. As an exception, in ATBC, low educational attainment corresponded to elementary or lower education, middle educational attainment to lower or upper secondary education, and high educational attainment to higher than upper secondary education. Based on smoking history, we categorised participants as never, former or current smokers. In ATBC, all participants were current smokers due to the study design. Leisure time physical activity was also assessed according to three categories: inactive (light activities, e.g., reading), somewhat active (e.g., walking, gardening) and active (competition and other heavy sports). Based on self-reported HRT use, we categorised women into ever (current or former use) and never users.

Participants’ weight (kg) and height (m) were measured at the health examination according to international standard protocols [34]. We calculated body mass index (BMI) by dividing weight by height squared (kg/m^2^).

### Statistical analyses

In descriptive analyses of baseline characteristics, we calculated medians and interquartile ranges (IQR) for continuous variables and proportions for categorical variables. We initially examined associations between CRC risk (hazard ratios [HR] and 95% confidence intervals [CI]) and the consumption of each food group used in the substitution analyses individually, both according to their consumption quintiles and per consumption of 100 g/week (red meat, whole grains, vegetables, fruits and legumes) or 50 g/week (processed meat). The associations were estimated using two-stage meta-analyses. We calculated cohort-specific HRs and 95% CIs for CRC risk by Cox proportional hazards multivariate models, after which we pooled the cohort-specific estimates, weighted by the inverse of their variances, using random-effects models [35]. Heterogeneity between the cohorts was tested by Q-statistics. In these analyses, we observed a direct association between legume consumption (100 g/week) and CRC risk. As our objective was to examine substitutions that could lower CRC risk, we excluded legumes from the substitution analyses (Online Resource 4).

We used a leave-one-out model to study partial substitutions of 100 g/week of red meat or 50 g/week of processed meat with corresponding amounts of whole grains, vegetables, fruits or a combination of these. The leave-one-out model was formed by including in the model the substitution variable (whole grains, vegetables, fruits or a combination of these) and a sum variable constructed of the substitution variable and the food that is being substituted (red meat or processed meat) (Online Resource 3) [36, 37]. The derived HRs and 95% CIs indicated the CRC risk following a parallel decrease in red or processed meat consumption and increase in the consumption of the substitution variable. The substituted amounts reflected the daily average consumption of red meat (100 g/week) and processed meat (50 g/week) in our study population. To consider the effect of the substituted amount, we conducted additional analyses by first doubling the red meat substitution to 200 g/week and processed meat substitution to 100 g/week and then further doubling the processed meat substitution to 200 g/week.

As sensitivity analyses, we modelled the substitutions excluding participants (1) who consumed red meat < 100 g/week or processed meat < 50 g/week or (2) who were diagnosed with CRC within the first two years of follow-up. We also studied interactions between the substitutions and sex, age (< median, ≥median), BMI (< 25, 25−<30, ≥ 30 kg/m^2^), HRT use (in women; never, ever) and mean follow-up time (< 15.3 years, ≥ 15.3 years) by including an interaction term in the analyses. Because the median whole grain intake in ATBC was very high, we also explored interactions in the substitutions with whole grains by the median whole grain intake in the total population (< 587 g/week vs. ≥587 g/week [84 g/d]). Finally, to consider the predomination of ATBC in terms of the number of participants and CRC cases, as well as other differences with the remaining cohorts (e.g., timing and background factors), we examined the substitutions separately in ATBC and the remaining cohorts.

In each analysis, we applied two sets of adjustments: age, sex and energy intake (model 1); and age, sex, energy intake, educational attainment, smoking habits, height, BMI, leisure time physical activity, HRT use (in women), and consumption of alcohol and dairy products (model 2). Additionally, in ATBC, we adjusted the substitution analyses for family history of CRC (information only available in ATBC). We chose the confounding factors based on the literature. All analyses were conducted using R statistical software version 3.6 [38]. The HRs were pooled by R package meta [39]. Statistical significance was determined based on 95% CIs and a two-tailed *P*-value < 0.05.

## Results

We present results from analyses of women and men combined because of a small number of CRC cases in women and because no interaction occurred between the sexes. Among the 43 788 participants, the median follow-up was 28.8 years (range 7.8 − 29.9 years), during which 1124 CRCs were diagnosed (Table 1). The median age at baseline ranged from 50 (Health 2000) to 60 years (HBCS) (Table 2). ATBC differed from the other cohorts by having a higher proportion of participants with low educational attainment (78% vs. 30 − 34%) and smokers (100% vs. 17 − 26%). The proportion of participants physically inactive in leisure time ranged from 19% (DILGOM 2007) to 42% (ATBC). Regarding dietary intake, participants in ATBC tended to consume more dairy products, alcohol, processed meat and whole grains, and fewer vegetables, fruits and legumes than participants in the other cohorts. For example, average processed meat consumption in ATBC was double that in HBCS (420 vs. 210 g/week), while vegetable consumption in ATBC was less than half the amount consumed in the other cohorts (658 vs. 1526 − 1848 g/week). Legume consumption was low in all cohorts (28 − 70 g/week).

**Table 2:** Baseline characteristics of the participants included in the pooled analyses by cohorts (medians and interquartile ranges [IQR] or percentages)

	ATBC	Health 2000	HBCS	DILGOM 2007	FINRISK 2012
Age, years	57 (8)	50 (22)	60 (4)	53 (22)	53 (24)
Low educational attainment^a^, %	78	33	34	30	33
Current smoker, %	100	26	24	18	17
Inactive in leisure time, %	42	28	31	19	20
Body mass index, kg/m^2^	26 (5)	26 (6)	27 (6)	26 (6)	26 (6)
HRT use (women), ever %	-	31	68	16	14
Energy, MJ/d	10.8 (4.0)	9.1 (3.9)	8.7 (4.0)	9.9 (4.5)	8.9 (4.1)
Dietary fibre, g/d	17 (13)	23 (13)	25 (14)	29 (16)	24 (14)
Alcohol (100%), g/d	11 (23)	2 (7)	5 (11)	4 (9)	4 (9)
Dairy products, g/d	699 (502)	546 (466)	436 (435)	584 (509)	579 (516)
Substitution variables					
Red meat, g/week	455 (273)	511 (343)	420 (350)	511 (406)	462 (357)
Processed meat, g/week	420 (406)	252 (336)	210 (280)	280 (350)	266 (343)
Whole grains^b^, g/week	700 (595)	406 (406)	378 (357)	532 (441)	483 (455)
Vegetables^c^, g/week	658 (588)	1526 (1295)	1722 (1449)	1848 (1533)	1589 (1323)
Fruits, g/week	756 (819)	1099 (1435)	1512 (1897)	1491 (1687)	1085 (1274)
Legumes, g/week	28 (35)	63 (63)	56 (63)	70 (70)	70 (70)

ATBC, the Alpha-Tocopherol, Beta-Carotene Cancer Prevention Study; Health 2000, the Health 2000 Health Examination Survey; HBCS, the Helsinki Birth Cohort Study; DILGOM 2007, the DIetary, Lifestyle and Genetic Determinants of Obesity and Metabolic Syndrome 2007 Study; FINRISK 2012, the National FINRISK 2012 Study; HRT, hormone replacement therapy

^a^In all cohorts except for ATBC, participants were categorised into those with low, middle or high educational attainment according to cohort-specific tertiles of self-reported school years, considering sex and birth year. In ATBC, low educational attainment corresponded to elementary or lower education.

^b^Whole grain intake was assessed based on the combined consumption of rye, oat and barley (32).

^c^Vegetables excluding legumes and potatoes and including nuts and seeds

Red meat consumption was associated with a 76% higher and processed meat consumption with a 26% higher CRC risk among participants in the highest consumption quintile (Q5) compared with those in the lowest quintile (Q1) (red meat: HR 1.76, 95% CI 1.05 − 2.94, *P*-trend = 0.041; processed meat: 1.26, 1.00 − 1.59, *P* = 0.026; model 2) (Online Resource 4). With the amounts used in the substitution analyses, both red meat (100 g/week; 1.03, 1.00 − 1.06, *P* = 0.027; model 2) and processed meat were associated with small increases in CRC risk, although for processed meat, the association was statistically significant only in model 1 (50 g/week; 1.01, 1.00 − 1.02, *P* = 0.027). We also observed a direct association between legume consumption and CRC risk (100 g/week; 1.14, 1.05 − 1.25, *P* = 0.003; model 2). Conversely, fruit consumption had an inverse association with CRC risk (100 g/week; 0.99, 0.98 − 1.00, *P* = 0.049; model 2). Otherwise, we observed no associations between the plant-based foods and CRC risk.

In the substitution analyses, we observed small decreases in CRC risk when 100 g/week of red meat was substituted with a corresponding amount of vegetables (0.97, 0.95 − 0.99, *P* = 0.008; model 2), fruits (0.97, 0.94 − 0.99, *P* = 0.007; model 2) or a combination of whole grains, vegetables and fruits (0.97, 0.95 − 0.99, *P* = 0.012; model 2) (Table 3). Similarly, we detected small risk reductions when processed meat (50 g/week) was substituted with vegetables (0.99, 0.98 − 1.00, *P* = 0.029, model 2) or fruits (0.99, 0.98 − 1.00, *P* = 0.036; model 2). Substituting red meat with whole grains and processed meat with the plant-based foods combined were also associated with a decreased CRC risk, but statistically significantly only in model 1. No notable heterogeneity occurred between the cohorts. Excluding participants who consumed red meat < 100 g/week or processed meat < 50 g/week attenuated the associations between the processed meat substitutions and CRC risk (*P*_*vegetables*_=0.06, *P*_*fruits*_=0.08; model 2). The results remained the same after we excluded participants diagnosed with CRC within the first two years of follow-up and, in ATBC, after we adjusted the analyses for family history of CRC (data not shown).

**Table 3:** Pooled associations between partial substitutions of red meat or processed meat with whole grains, vegetables, fruits, or a combination of these and colorectal cancer risk (hazard ratios [HR] and 95% confidence intervals [CI])

	Model 1^a^ HR (95% CI)	P	Model 2^b^ HR (95% CI)	P	P_het_.^c^
Substitution of red meat (100 g/week) with					
Whole grains^d^, 100 g/week	0.94 (0.89, 1.00)	0.047	0.96 (0.91, 1.01)	0.12	0.22
Vegetables^e^, 100 g/week	0.97 (0.94, 0.99)	0.004	0.97 (0.95, 0.99)	0.008	0.67
Fruits, 100 g/week	0.96 (0.93, 0.99)	0.002	0.97 (0.94, 0.99)	0.007	0.48
Whole grains, vegetables and fruits, 100 g/week	0.96 (0.94, 0.99)	0.004	0.97 (0.95, 0.99)	0.012	0.44
Substitution of processed meat (50 g/week) with					
Whole grains^d^, 50 g/week	0.98 (0.96, 1.01)	0.11	0.99 (0.98, 1.00)	0.25	0.73
Vegetables^e^, 50 g/week	0.99 (0.98, 1.00)	0.033	0.99 (0.98, 1.00)	0.029	0.81
Fruits, 50 g/week	0.99 (0.98, 1.00)	0.016	0.99 (0.98, 1.00)	0.036	0.80
Whole grains, vegetables and fruits, 50 g/week	0.99 (0.98, 1.00)	0.035	0.99 (0.98, 1.00)	0.07	0.88

^a^Model 1 was adjusted for sex, age (years, continuous) and energy intake (kJ/day, continuous).

^b^Model 2 was adjusted for variables in model 1^a^ + educational attainment (low, middle, high), smoking habits (never, former, current), height (m, continuous), body mass index (kg/m^2^, continuous), leisure-time physical activity (inactive, somewhat active, active), hormone replacement therapy use (in women; never, ever), and consumption of alcohol (100%; g/day, continuous) and dairy products (g/day, continuous).

^c^*P* for heterogeneity between the pooled cohorts was tested by Q-statistics (model 2).

^d^Whole grain intake was assessed based on the combined consumption of rye, oat and barley (32).

^e^Vegetables excluding legumes and potatoes and including nuts and seeds

After excluding ATBC from the cohorts, we observed a statistically significant 7% decrease in CRC risk when red meat was substituted with whole grains (0.93, 0.87 − 0.99, *P* = 0.021; model 2). Excluding ATBC also slightly strengthened the inverse associations between CRC risk and substituting red meat with vegetables (0.96, 0.93 − 1.00, *P* = 0.028; model 2), fruits (0.95, 0.92 − 0.98, *P* = 0.002; model 2) or the plant-based foods combined (0.97, 0.93 − 0.99, *P* = 0.004; model 2) (Online Resource 5). The associations between the processed meat substitutions and CRC risk remained essentially the same, although the 1% risk reductions following the substitutions with vegetables or fruits were no longer statistically significant.

Doubling the substitutions to 200 g/week for red meat and 100 g/week for processed meat resulted in somewhat larger risk reductions. Substituting red meat with vegetables, fruits, or the plant-based foods combined reduced CRC risk by 6%, 7% and 6%, respectively (vegetables: 0.94, 0.89 − 0.98, *P* = 0.008; fruits: 0.93, 0.89 − 0.98, *P* = 0.007; the plant-based foods combined: 0.94, 0.89 − 0.99, *P* = 0.012; model 2). For processed meat, the substitutions with vegetables or fruits resulted in borderline 2% risk reductions. After increasing the processed meat substitution to 200 g/week, these risk reductions increased to 4% (vegetables: 0.96, 0.92 − 1.00, *P* = 0.029; fruits: 0.96, 0.93 − 1.00, *P* = 0.036; model 2).

We observed no interactions between the substitutions (red meat: 100 g/week; processed meat: 50 g/week) and sex, age, BMI, HRT use, or follow-up time (data not shown). When the substitutions with whole grains were examined separately for those with < median or ≥ median whole grain intake, we detected significant interactions for both red meat and processed meat (*p*_interaction_=0.001). Among participants with < median whole grain intake, substituting red meat or processed meat with whole grains was associated with 8% and 4% decreases in CRC risk, respectively (red meat: 0.92, 0.86 − 0.98; processed meat: 0.96, 0.93 − 0.99; model 2), while no statistically significant associations occurred among those with ≥ median whole grain intake (red meat: 0.99, 0.94 − 1.05; processed meat: 1.00, 0.99 − 1.01; model 2).

## Discussion

In this pooled analysis of five large Finnish cohorts, substitutions of 100 g/week of red meat or 50 g/week of processed meat with a corresponding amount of vegetables or fruits were associated with small decreases in CRC risk. We observed no association between substitutions of red or processed meat with whole grains and CRC risk in the total population. However, among participants with < median whole grain intake, substitutions with whole grains resulted in up to 8% reduction in CRC risk.

Substitutions of red meat or processed meat with whole grains, vegetables or fruits have not been examined previously in relation to CRC risk. In a study exploring *protein* substitutions within a large US cohort (*n* = 490 000), no association was observed between replacing protein from red meat with protein from vegetables and fruits and CRC risk [20]. Similarly, in an Italian study (*n* = 45 000), no association was observed between replacing 3% of energy intake from animal protein with plant-based protein from low glycaemic index foods (pasta, vegetables, fruits and legumes) and the risk of colon or rectal cancer.

In this study, the associations observed between the substitutions and CRC risk were modest. We had expected to detect stronger associations, particularly regarding processed meat substitutions, given the compelling evidence in the literature on the carcinogenicity of processed meat consumption [6]. Overall, the modest results are likely attributed to the nonsignificant or small associations between the food groups individually and CRC risk; consumption of processed meat (50 g/week), whole grains (100 g/week) and vegetables (100 g/week) had no statistically significant association with CRC risk, whereas fruit consumption (100 g/week) had a small inverse association and red meat consumption (100 g/week) a small direct association with CRC risk. Regardless, the substitutions with the plant-based foods appeared to strengthen the associations observed between red or processed meat consumption and CRC risk.

We observed a reduction in CRC risk when red meat or processed meat was partially substituted with vegetables or fruits. The beneficial effects of vegetables and fruits on CRC risk may arise from their high content of dietary fiber and polyphenols [40]. Indeed, fibre has been consistently associated with lower CRC risk; its protective effects seem to be linked to its ability to increase stool volume and decrease transit time, as well as to its fermentation in the gut into short-chain fatty acids with anticarcinogenic properties [41, 42]. The protective effects of polyphenols have been linked to their anti-inflammatory, antioxidant and pro-apoptotic features [43]. Nevertheless, when examined individually, we did not detect any association between vegetable consumption and CRC risk. This diverges from the WCRF/AICR meta-analysis, in which a small inverse association was observed for the consumption of 100 g/d of vegetables in 11 studies [13]. The association was, however, driven by one study, while most studies in the meta-analysis reported null associations. When fruit consumption was examined individually, we detected a small inverse association with CRC risk. In the WCRF/AICR meta-analysis, no association was observed in 13 studies, whereas another meta-analysis of 19 prospective studies reported a 3% reduction in CRC risk per 100 g/d increase in fruit consumption [40]. As uncertainty remains in the evidence regarding both vegetables and fruits, more research is needed on the substitutions as well as their individual associations with CRC risk.

Contrary to our expectations, neither whole grain intake nor the partial substitution of red meat or processed meat with whole grains was associated with CRC risk. This is discordant with the consistent evidence in the literature of an inverse association between whole grain intake and CRC risk. The non-significant results in our study seemed to be linked to ATBC because after excluding it from the cohorts, we observed a statistically significant 7% decrease in CRC risk when red meat was substituted with whole grains. We hypothesized that this discrepancy could be attributed to the high whole grain intake in ATBC (700 g/week [100 g/d]). Therefore, we stratified the analysis by the median whole grain intake in the total population (587 g/week [84 g/d]). Following this, the substitutions of red or processed meat with whole grains were not statistically significantly associated with CRC risk among participants with ≥ median whole grain intake, whereas, among participants with < median whole grain intake, the substitutions were associated with up to 8% reduction in CRC risk. This risk reduction is the largest observed in our study for either red meat or processed meat. Moreover, as average whole grain intake levels of adults fall below 84 g/d in Finland (60 g/d) [32] and in many other Western countries, such as Denmark (69 g/d) [44], the UK (median 20 g/d) [45], Italy (4 g/d) [46], Australia (21 g/d) [47] and the US (16 g/d) [48], this result suggests that most adults in these countries would benefit from the substitution with whole grains. The non-significant association among those with higher than median whole grain intakes could arise from a plateau effect in health benefits after achieving a certain whole grain intake level. Nevertheless, evidence from meta-analyses does not currently support a non-linear association between whole grain intake and CRC [15, 49]. Thus, more research is needed to better understand this phenomenon.

We did not model substitutions of red meat or processed meat with legumes because, in our study population, legume consumption of 100 g/week seemed to have a direct association with CRC risk. This finding was unexpected as in a recent meta-analysis of 29 case-control and cohort studies, a 100 g/d increase in legume consumption was associated with a 21% lower CRC risk [50]. The direct association in our study is likely related to low legume consumption in the cohorts (28 − 70 g/week). The association could also be linked to other dietary components that legumes have traditionally been consumed with; for example, pea soup, which traditionally includes pork, is among the most frequently consumed legume-based dishes in Finland. Overall, the observed association should be interpreted cautiously.

We could have detected more substantial risk reductions by modelling daily rather than weekly substitutions. Nevertheless, as we aimed to investigate substitutions that would be easy to implement and maintain in real life, substituting all or most daily red or processed meat consumption would not have met these criteria. As regards real-life implications, within our study population, the modelled substitutions correspond to approximately one and a half days’ portion of red meat (100 g) and one day’s portion of processed meat (50 g) in a week. On a food level, 100 g of red meat approximately equals one small beef steak (cooked) and 50 g of processed meat half a sausage or 3 − 4 cold cuts. Regarding the plant-based substitutes, a 50 − 100 g portion corresponds, for whole grains, to 3 − 6 slices of whole grain bread or 1 − 2 big portions of cooked whole grain pasta; for vegetables 0.5 − 1 small portion of oven-roasted vegetables; and for fruits 0.5 − 1 small apple or (peeled) banana. As these examples demonstrate, the modelled substitutions were very moderate and would be achievable to most people. Moreover, even if the substitutions were doubled, the dietary changes would remain feasible for most.

Although the observed risk reductions were small, we consider the results encouraging; even small population-level changes towards more plant-based diets could reduce the CRC burden in the population. These findings support the implementation of public health strategies to promote more plant-based diets as part of CRC primary prevention. Moreover, on an individual level, the knowledge that already small changes towards more plant-based diets can benefit health might encourage a gradual transition to healthier and more sustainable diets.

The main strength of this study is the large, pooled study population from five Finnish cohorts with long median follow-up and comprehensive information on participants’ diet, lifestyle and health. Another key strength is the use of comprehensive and reliable cancer data from a nationwide cancer registry, which resulted in nearly complete case ascertainment in each cohort. Furthermore, we assessed dietary intake in each cohort with a validated FFQ, which is a widely accepted and commonly used dietary assessment method in epidemiological research.

This study also has limitations. Because the FFQ is based on self-reporting, it may expose the data to reporting biases (e.g., under- or overreporting) and misclassification. To diminish these effects, we adjusted the analyses for energy intake. Another limitation is the use of a single baseline measurement, for which we could not consider potential changes in food consumption over the follow-up. Regarding CRC, we did not analyse the subtypes (proximal and distal colon cancer, rectal cancer) separately owing to the low number of, especially, distal colon cancers. This is a limitation, as the subtypes may have different risk factors. Even though we adjusted the analyses for several key dietary and lifestyle factors, we cannot rule out residual confounding from other unmeasured factors (e.g., genetic factors). In the substitution analyses, we did not model the substitutions in an isocaloric manner but according to corresponding consumption quantities. Therefore, despite the adjustment for energy intake, the substitutions have likely resulted in residual differences in energy intake, which would be reflected in the consumption of other foods in the diet. Nevertheless, as the modelled substitutions were very moderate and on a weekly level, we presumed that the variation in energy intake would remain on a level that would not considerably change the participants’ nutritional profiles; even if the residual energy would be consumed as other foods considered unhealthy, such as sodas or pastry products, increase in their consumption would remain small. Finally, because of the more selected study population in ATBC (male smokers), the results may not be fully generalizable to the general adult population. For example, residual confounding by smoking likely remains. We were not able to examine the effect of smoking on our results by stratifying the substitution analyses by smoking status due to the low number of CRC cases in the remaining cohorts. Nonetheless, the adjustment for smoking in the remaining cohorts did not appear to have a notable effect on the results, and, apart from the substitutions with whole grains, the differences in the results between ATBC and the remaining cohorts were small. Moreover, we observed no heterogeneity between the cohorts. The results also appeared similar for women and men.

To conclude, our results suggested that even small, easily implemented partial substitutions of especially red meat, but also processed meat, with whole grains, vegetables, fruits or a combination of these could lower CRC risk in Finnish adults. These findings support the necessity of dietary shifts towards more plant-based diets for health and environmental reasons. More evidence is, however, required in real-life settings and different populations to better understand the implications of these substitutions on CRC risk. Furthermore, public health strategies to promote and support these dietary changes as a part of CRC primary prevention require more attention.

### Electronic supplementary material

Below is the link to the electronic supplementary material.

Supplementary Material 1

## Data Availability

The datasets used in this research are available upon request through the Findata permit procedure at https://www.findata.fi/en/.
