# Supplementary material for: Partial substitution of red meat or processed meat with plant-based foods and the risk of colorectal cancer
Source: Eur J Epidemiol. 2024 Jan 23;39(4):419–28. doi: 10.1007/s10654-024-01096-7 (PMC11101510; doi:10.1007/s10654-024-01096-7)
Supplement: Supplementary file 1 — Supplementary Material 1 [file 10654_2024_1096_MOESM1_ESM.pdf]

Supplementary material

Title: Partial substitution of red meat or processed meat with plant-based foods and the risk of colorectal cancer

Journal: European Journal of Epidemiology

Authors: Rilla Tammi*, Niina E Kaartinen, Kennet Harald, Mirkka Maukonen, Heli Tapanainen, Stephanie A Smith-Warner, Demetrius Albanes, Johan G Eriksson, Pekka Jousilahti, Seppo Koskinen, Maarit A Laaksonen, Sanna Heikkinen, Janne Pitkäniemi, Anne-Maria Pajari, Satu Männistö

*Corresponding author:

Finnish Institute for Health and Welfare (THL), P.O. Box 30, 00271, Helsinki, Finland

rilla.tammi@thl.fi

This supplementary material includes five Online Resources: (1) a flow chart showing the exclusions made in each cohort used in this study, (2) a table of foods included within each food group used in the substitution analyses, (3) the formula for a leave-one-out model used to model partial substitutions of red or processed meat with plant-based foods in relation to CRC risk, (4) a table of associations between the substitution variables and colorectal cancer risk continuously and per 100 g/week or 50 g/week (processed meat) consumption, and (5) a table of associations between partial substitutions of red or processed meat with plant-based foods and CRC risk displayed separately for ATBC and the remaining cohorts.

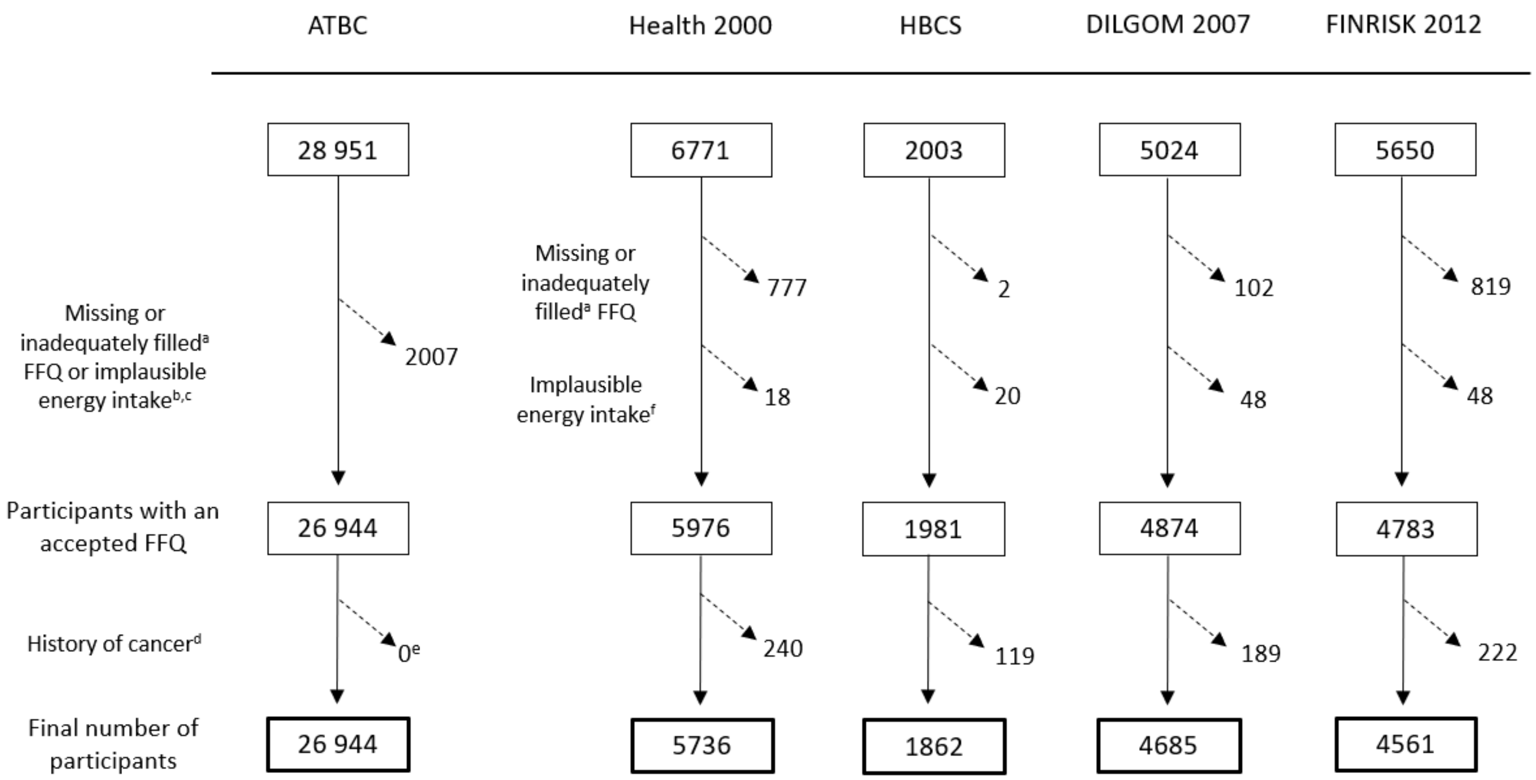

Online Resource 1 Exclusions and final study samples by cohort

ATBC, the Alpha-Tocopherol, Beta-Carotene Cancer Prevention Study; Health 2000, the Health 2000 Health Examination Survey; HBCS, the Helsinki Birth Cohort Study; DILGOM 2007, the DIetary, Lifestyle and Genetic Determinants of Obesity and Metabolic Syndrome 2007 Study; FINRISK 2012, the National FINRISK 2012 Study; FFQ, food frequency questionnaire

[a]Incomplete questionnaire with several empty food item rows (exclusions made case by case)

[b]Energy intake <1000 or >5000 kcal/d

[c]Due to differences in data gathering and reporting, in ATBC, the number of participants excluded for missing or inadequately filled FFQ or implausible energy intake are presented together.

[d]History of cancer other than non-melanoma skin cancer

[e]Individuals with a cancer history (other than non-melanoma skin cancer) were excluded in recruiting participants for the trial.

[f]Health 2000: energy intake <600 or >7000 kcal/d; HBCS, DILGOM 2007 and FINRISK 2012: 0.5% sex-specific extremes in energy intake distribution

Online Resource 2 Foods included in each food group used in the substitution analyses

Food group[a]	Included foods
Red meat	Beef, pork and lamb (e.g., minced meet, beef steak)
Processed meat	Sausages (e.g., fresh sausages, frankfurters, bratwurst) and cold cuts (e.g., smoked ham, meat cuts, meat sausages) [b]
Whole grains	Rye, oat and barley (e.g., rye flour, rolled oats) [c]
Vegetables	Cabbages, leaf vegetables, nuts and seeds[d], mushrooms, onions, root vegetables[e] and vegetable fruits
Fruits	Fruits (e.g., citrus fruits, apples) and berries (e.g., strawberry, blueberry)
Legumes	Beans, green peas, green beans and soya

[a]The food groups were defined based on the food classifications of the Finnish Food Composition Database Fineli® (31).
[b]Including processed meat made of beef, pork and lamb
[c]Rye, oat and barley combined has been shown to correspond well (r=0.99) to total whole grain intake among Finnish adults (32).
[d]Consumption of nuts and seeds is and has been very low in Finland, for which they were not considered as their own food group but included in vegetables.
[e]Excluding potatoes

Online Resource 3 The leave-one-out model for studying a partial substitution of red or processed meat with plant-based foods in relation to colorectal cancer (CRC) risk

Model expression[a]	Variables
$f(Y) = \alpha_1 A + \alpha_2(A + B)$ + confounders	$\alpha_1 A$ = beta coefficient[b,c] for the substitution variable (whole grains, vegetables, fruits, or a combination of these) by 100 g/week or 50 g/week consumption<br>$\alpha_2(A + B)$ = beta coefficient[b] for the sum variable constructed of the substitution variable and the food that is being substituted (red meat or processed meat) by total consumption

[a]Modified from Song & Giovannucci 2018 (36)
[b]Beta coefficients are calculated by Cox proportional hazards multivariate models.
[c]Parameter for the substitution effect. Beta coefficient for A (α_1) is exponentiated to calculate the hazard ratio (HR).

Online Resource 4 Pooled associations between consumption of the substitution variables (quintiles and 100 g/week or 50 g/week) and colorectal cancer risk (hazard ratios [HR] and 95% confidence intervals [CI])

	Quintile 1	Quintile 3	Quintile 5	P_{trend}	P_{het}[a]	100 g/week[b]	*P*
Red meat							
Median (IQR), g/week	234 (97)	467 (56)	860 (289)				
Colorectal cancer cases, n	195	231	238				
Model 1[c]	1.00	1.14 (0.83, 1.57)	1.91 (1.10, 3.29)	0.033	0.06	1.04 (1.01, 1.07)	0.009
Model 2[d]	1.00	1.01 (0.83, 1.23)	1.76 (1.05, 2.94)	0.041	0.11	1.03 (1.00, 1.06)	0.027
Processed meat							
Median (IQR), g/week	107 (72)	359 (71)	916 (417)				
Colorectal cancer cases, n	161	240	263				
Model 1[c]	1.00	1.15 (0.93, 1.42)	1.31 (1.04, 1.64)	0.011	0.99	1.01 (1.00, 1.02)	0.027
Model 2[d]	1.00	1.14 (0.92, 1.42)	1.26 (1.00, 1.59)	0.026	1.00	1.01 (1.00, 1.02)	0.1
Whole grains[e]							
Median (IQR), g/week	166 (127)	587 (93)	1233 (392)				
Colorectal cancer cases, n	196	194	287				
Model 1[c]	1.00	0.73 (0.60, 0.90)	0.71 (0.44, 1.14)	0.22	0.34	0.97 (0.93, 1.02)	0.27
Model 2[d]	1.00	0.76 (0.62, 0.93)	0.74 (0.43, 1.26)	0.33	0.39	0.99 (0.95, 1.03)	0.47
Vegetables[f]							
Median (IQR), g/week	314 (165)	911 (184)	2481 (1247)				
Colorectal cancer cases, n	249	278	119				
Model 1[c]	1.00	1.16 (0.97, 1.38)	0.86 (0.61, 1.21)	0.28	0.25	1.00 (0.99, 1.01)	0.78
Model 2[d]	1.00	1.13 (0.94, 1.35)	0.90 (0.54, 1.48)	0.26	0.12	1.00 (0.99, 1.02)	0.52
Fruits							
Median (IQR), g/week	215 (182)	884 (187)	2389 (1239)				
Colorectal cancer cases, n	247	260	151				
Model 1[c]	1.00	1.15 (0.76, 1.73)	0.82 (0.66, 1.03)	0.12	0.21	0.99 (0.98, 1.00)	0.018
Model 2[d]	1.00	1.31 (0.78, 2.22)	0.82 (0.65, 1.03)	0.08	0.65	0.99 (0.98, 1.00)	0.049
Legumes							
Median (IQR), g/week	9 (11)	38 (10)	121 (86)				
Colorectal cancer cases, n	257	243	154				
Model 1[c]	1.00	1.07 (0.90, 1.28)	1.02 (0.79, 1.30)	0.06	0.12	1.13 (1.04, 1.23)	0.006

Model 2[d]	1.00	1.06 (0.89, 1.27)	0.99 (0.77, 1.28)	0.06	0.04	1.14 (1.05, 1.25)	0.003

IQR, interquartile range

[a]*P* for heterogeneity between the pooled cohorts was tested by Q-statistics (model 2).

[b]50 g/week for processed meat

[c]Model 1 was adjusted for sex, age (years, continuous) and energy intake (kJ/day, continuous).

[d]Model 2 was adjusted for variables in model 1[c] + educational attainment (low, middle, high), smoking habits (never, former, current), height (m, continuous), body mass index (kg/m^2, continuous), leisure-time physical activity (inactive, somewhat active, active), hormone replacement therapy use (in women; never, ever), and consumption of alcohol (100%; g/day, continuous) and dairy products (g/day, continuous)

[e]Whole grain intake was assessed based on the combined consumption of rye, oat and barley (32).

[f]Vegetables excluding legumes and potatoes and including nuts and seeds

Online Resource 5 Associations between partial substitutions of red meat or processed meat with whole grains, vegetables, fruits or a combination of these and colorectal cancer risk in ATBC and the remaining cohorts (hazard ratios [HR] and 95% confidence intervals [CI])

	ATBC		Remaining cohorts[a]		
	HR (95%CI)[b]	*P*[b]	HR (95%CI)[b]	*P*[b]	$P_{het.}$[b,c]
Substitution of red meat (100 g/week) with					
Whole grains[d], 100 g/week	0.99 (0.96, 1.03)	0.73	0.93 (0.87, 0.99)	0.021	0.56
Vegetables[e], 100 g/week	0.97 (0.94, 1.01)	0.12	0.96 (0.93, 1.00)	0.028	0.53
Fruits, 100g/week	0.98 (0.95, 1.01)	0.29	0.95 (0.92, 0.98)	0.002	0.74
Whole grains, vegetables and fruits, 100 g/week	0.99 (0.96, 1.02)	0.32	0.97 (0.93, 0.99)	0.004	0.62
Substitution of processed meat (50 g/week) with					
Whole grains[d], 50 g/week	1.00 (0.99, 1.01)	0.46	0.97 (0.94, 1.01)	0.14	0.91
Vegetables[e], 50 g/week	0.99 (0.98, 1.00)	0.031	0.99 (0.97, 1.02)	0.58	0.71
Fruits, 50 g/week	0.99 (0.98, 1.00)	0.07	0.99 (0.96, 1.01)	0.30	0.67
Whole grains, vegetables and fruits, 50 g/week	0.99 (0.98, 1.00)	0.09	0.99 (0.97, 1.02)	0.47	0.75

ATBC, the Alpha-Tocopherol, Beta-Carotene Cancer Prevention Study

[a]the Health 2000 Health Examination Survey (Health 2000), the Helsinki Birth Cohort Study (HBCS), the DIetary, Lifestyle and Genetic Determinants of Obesity and Metabolic Syndrome 2007 Study (DILGOM 2007), the National FINRISK 2012 Study (FINRISK 2012)

[b]Model 2 was adjusted for sex, age (years, continuous), energy intake (kJ/day, continuous), educational attainment (low, middle, high), smoking habits (never, former, current), height (m, continuous), body mass index (kg/m^2, continuous), leisure-time physical activity (inactive, somewhat active, active), hormone replacement therapy use (in women; never, ever), and consumption of alcohol (100%; g/day, continuous) and dairy products (g/day, continuous)

[c]*P* for heterogeneity between the pooled cohorts was tested by Q-statistics (model 2).

[d]Whole grain intake was assessed based on the combined consumption of rye, oat and barley (32).

[e]Vegetables excluding legumes and potatoes and including nuts and seeds.
